# Correction: AQP4-Dependent Water Transport Plays a Functional Role in Exercise-Induced Skeletal Muscle Adaptations

**DOI:** 10.1371/annotation/86fc2632-913c-490d-8b9b-e925b38baec5

**Published:** 2013-06-18

**Authors:** Davide Basco, Bert Blaauw, Francesco Pisani, Angelo Sparaneo, Grazia Paola Nicchia, Maria Grazia Mola, Carlo Reggiani, Maria Svelto, Antonio Frigeri

The legends for Figures 2 through 8 are incorrect. The figure under which the legend begins "Figure 3..." is Figure 2, the figure under which the legend begins "Figure 4..." is Figure 3, the figure under which the legend begins "Figure 5..." is Figure 4, the figure under which the legend begins "Figure 6..." is Figure 5, the figure under which the legend begins "Figure 7..." is Figure 6, the figure under which the legend begins "Figure 8..." is Figure 7, and the figure under which the legend does not begin with a numeric label is Figure 8. 

